# Post-traumatic Stress Disorder in Veterans: A Concept Analysis

**DOI:** 10.3390/bs14060485

**Published:** 2024-06-07

**Authors:** Tormechi Chambliss, Jung-Lung Hsu, Mei-Lan Chen

**Affiliations:** 1 School of Nursing, Byrdine F. Lewis College of Nursing and Health Professions, Georgia State University, Atlanta, GA 30303, USA; tchambliss4@student.gsu.edu; 2 Department of Neurology, New Taipei Municipal TuCheng Hospital (Built and Operated by Chang Gung Medical Foundation), New Taipei City 236, Taiwan; tulu@ms36.hinet.net

**Keywords:** post-traumatic stress disorder, PTSD, DSM-5, veterans, concept analysis, trauma, defining attributes, antecedents, empirical referents

## Abstract

Post-traumatic stress disorder (PTSD) occurs when an individual experiences a traumatic event that exceeds the limits of psychological endurance. Many veterans experience PTSD. PTSD can negatively impact veterans’ quality of life, functioning, life satisfaction, and overall well-being. It is important to analyze the concept of PTSD in the veteran population. This concept analysis aimed to investigate the defining attributes, a model case, antecedents, consequences, and empirical referents related to the concept of PTSD among veterans. Walker and Avant’s method was used to guide this concept analysis of PTSD. The results showed that three attributes were determined from the analysis: intrusive memories of traumatic events, feelings of isolation and estrangement, and negative cognitions. PTSD is conceptualized as a collection of symptoms that arise from highly traumatic experiences. The military environment predisposes veterans to traumatic events that should be identified or acknowledged. A better understanding of the concept of PTSD can facilitate the development of effective interventions for the veteran population and enhance their mental health.

## 1. Introduction

Post-traumatic stress disorder (PTSD), a mental health disorder, occurs when an individual experiences a trauma or traumatic event that exceeds the limits of his or her own psychological endurance [1]. PTSD has also been termed using other names such as “shell shock”, “combat fatigue”, and “post-Vietnam syndrome” [2]. Originally developed in 1952, the Diagnostic and Statistical Manual of Mental Disorders (DSM) was developed in response to many World War II (WWII) soldiers who described heightened levels of psychological stress due to their experiences in combat [3]. PTSD was not officially recognized as a diagnosis until 1980 with the publication of the DSM-III [3].

The fifth edition of the DSM (DSM-5) divided PTSD symptoms into four clusters: intrusion symptoms, avoidance, negative alterations in cognitions and mood, and alterations in arousal and reactivity [4]. It highlighted changes related to how PTSD was diagnosed, making it no longer an anxiety disorder, but placing it under the category of trauma–stressor-related disorders [3]. The DSM-5 is used in the military mostly for the purposes of determining eligibility for disability or fitness for duty [3]. The DSM-5 states that an individual must meet eight criteria to be diagnosed with PTSD [5]. One advantage of using the DSM-5 in military veterans is that it increased the number of PTSD symptoms from 17 to 20, which included mood and cognition manifestations to trauma [3]. Despite this increase in trauma-related experiences, many will still not meet the eight criteria that are required. For example, criterion C states an individual must have trauma-related thoughts and feelings or trauma-related reminders; without one of these avoidance symptoms, there cannot be a diagnosis of PTSD [3]. In 2022, a more updated version of the DSM-5, the DSM-5-TR, was published. No changes were made to the diagnostic criteria for PTSD [6].

PTSD results in significant distress in sufferers [1] and is associated with significant physical and psychological consequences [7]. A higher prevalence of mental health conditions, such as PTSD, is observed among veterans. The prevalence of PTSD in Vietnam veterans ranges from 18.7% to 30.9% during their lifetime [8]. The overall lifetime prevalence of PTSD in US veterans is 7% compared to US civilians at 6% [9]. If war is a traumatic stressor, the prevalence among surviving US veterans is as high as 80%, as veterans who are employed in war zones are more likely to have PTSD than veterans who are not [9,10,11]. More than one million US veterans have a service-connected disability for PTSD [12]. PTSD is a significant issue among veterans due to its negative impact on their mental health, quality of life, and overall well-being [13]. The debilitating symptoms of PTSD lead to impaired daily functioning and interpersonal relationships [13]. Moreover, veterans have a lower rate of treatment initiation and engagement [14]. Although services are available, most veterans do not access them [15]. The stigma surrounding mental health in the military can prevent veterans from seeking the necessary care, exacerbating the severity of the condition [3,16]. As a result of barriers to seeking treatment, there is significant morbidity and mortality associated with them that include depression, substance abuse, and suicide [17]. These barriers include lack of knowledge and issues with the therapist, the staff, and the healthcare system [18,19,20,21]. Reducing barriers to treatment seeking may be possible by understanding what constitutes this debilitating disease. 

It is important to understand and be educated on how PTSD can affect veterans [22]. Also, PTSD has a dramatic influence on not just the veteran, but on social and family relationships, and these relationships can either facilitate or hinder recovery [23]. However, the diagnostic criteria for PTSD are complex and ambiguous, and their complexity is attributed to the disease [24]. The DSM-5 is beneficial as it provides a set of criteria for diagnosing PTSD; however, a concept analysis offers a more comprehensive and deeper understanding of the disease and can foster more effective approaches to assessment, treatment, and support. With limitations being on the DSM-5 diagnosing criteria for veterans with PTSD, a concept analysis can help clarify any ambiguities beyond the diagnostic criteria [3,4,5,11,24]. Therefore, this study aimed to analyze the concept of PTSD in the veteran population. 

## 2. Walker and Avant’s Concept Analysis Method

A simplified version of Wilson’s concept analysis by Walker and Avant [25] was used as a guide for this concept analysis. Instead of Wilson’s 11 steps, Walker and Avant [25] modified it to 8 steps. Walker and Avant’s concept analysis method provides a structured approach that offers clear guidelines for identifying, defining, and clarifying concepts. It also enables the practical application of findings to research, education, and clinical practice [26]. The steps are as follows: (1) selecting a concept, (2) determining the aim or purpose of the analysis, (3) identifying the uses of the concept, (4) determining the defining attributes, (5) identifying the model case, (6) identifying additional cases, (7) identifying antecedents and consequences, and (8) defining empirical referents [25].

The following sections will demonstrate the findings of this concept analysis of PTSD in veterans using Walker and Avant’s method.

## 3. Uses of PTSD

Identifying the usage of a concept is essential in confirming its defining attributes [25,26]. Not recognizing the application of a concept could lead to an analysis that is restrictive of the outcome’s effectiveness. Therefore, using thesauruses, dictionaries, or other available literature can aid in manifesting the utilization of a concept. The Merriam-Webster Online Dictionary defines PTSD as “a psychological reaction occurring after experiencing a highly stressful event that is usually characterized by depression, anxiety, flashbacks, recurrent nightmares, and avoidance of reminders of the event” [27]. The Oxford Learner’s Dictionaries defines PTSD as “a mental illness following a very frightening or shocking experience, which usually involves feeling anxious and depressed and having frightening thoughts” [28]. The American Heritage Medical Dictionary defines PTSD as “a psychiatric disorder resulting from a traumatizing experience, such as torture, rape, or military combat, characterized by recurrent flashbacks of the traumatic event, nightmares, persistent negative emotions such as anger, fear, or shame, and difficulty experiencing positive emotions” [29]. The American Psychological Association Dictionary of Psychology defines PTSD as “a disorder that may result when an individual lives through or witnesses an event in which he or she believes that there is a threat to life or physical integrity and safety and experiences fear, terror, or helplessness” [30]. According to the US Department of Veterans Affairs [5], PTSD is a mental health problem that certain people will develop after being exposed to or witnessing a life-threatening event. PTSD will develop if the nature of the trauma experienced is a significant factor in a person’s life.

## 4. Defining Attributes

Walker and Avant describe defining attributes as those that are most frequently linked with the concept [25]. The defining attributes of PTSD are intrusive memories of traumatic events, feelings of isolation and estrangement, and negative cognitions.

### 4.1. Intrusive Memories of Traumatic Events

Veterans with PTSD often have episodes of flashbacks or nightmares due to intrusive memories of traumatic events. Re-experiencing or reliving memories is a hallmark sign of PTSD [31]. This leads to unwanted upsetting memories and emotional distress [5]. Any internal or external reminders of traumatic events can trigger re-experiencing symptoms. The intense, disturbing thoughts and feelings that are related to the traumatic event can last long after the event has ended, while feelings of estrangement and avoidance are heightened due to the strong negative reactions from intrusive memories [32].

### 4.2. Feelings of Isolation and Estrangement

Veterans with PTSD tend to avoid trauma-related situations, people, and places because of the symptom of avoidance. It is common for them to suffer long-term interpersonal and social adversities that leave them feeling hopeless [33]. In the context of this effortful avoidance, individuals are unable to experience positive feelings and face challenges in trusting others [14]. In order to avoid other PTSD symptoms that often coincide with one another, those who experience PTSD often engage in avoidance behaviors [31]. Their efforts to avoid reliving the trauma will include avoiding people, places, and even conversations. Symptoms of recurrent experiences are associated with this. When veterans are exposed to trauma, they feel alone and defenseless, and once they are at home, feelings of isolation and estrangement are heightened [10].

### 4.3. Negative Cognitions

When veterans have negative cognitions after they experience a traumatic event, it is a predictor in the development of PTSD. They attempt to integrate experiences with their existing beliefs, which leads to self-blame and negative beliefs about oneself if the integration is unsuccessful [34]. People with PTSD tend to have negative thoughts and feelings about themselves, others, and the world, and begin to lose their individual perspectives [11,34,35]. Having a sense of oneself in the world allows for feelings of connectedness, which is associated with mental wellness [36]. They often have difficulty trusting people and find it hard to be happy. Their perceptions are distorted and what once seemed normal is no longer the case for the person dealing with PTSD. Veterans are at an increased risk of suicide, which can be explained by PTSD and the negative beliefs they have about themselves [34].

## 5. Model Case and Additional Cases

### 5.1. Model Case

A model case demonstrates all the defining attributes of a concept [25].

S.C. was recently medically discharged from the United States Army. He served in the Iraq war and fought on the front lines. He experienced many traumatic situations while in war where he had to defend himself and his team against enemy trespassers. Once returning home, he had a difficult time in readjusting to civilian life. He struggled with being around people as he did not know who he could trust and began to isolate himself at home. He did not like the feeling of always being on guard just in case someone was waiting to attack him. While in this alone state, he began to have negative thoughts about himself and intrusive memories of the traumatic event. He felt as if he was not worthy to be around people based on the things he experienced in the war. He began to blame himself for the way he was feeling. 

This is a model case because it displays all of the previously discussed attributes of PTSD. Because of the traumatic event, S.C. began to have intrusive memories of the traumatic event, isolate himself from others, and have negative thoughts about himself.

### 5.2. Borderline Case

A borderline case allows us to see why the model case is consistent with the concept of interest [25]. It exhibits most of the defining attributes, but not all of them. 

C.D. is a veteran of the United States Army who completed two tours in Iraq. He has had many traumatic experiences with firing his weapon. He has been discharged from the military for two years. He works at a military base in his hometown where he is the first commander of his team. He spends time with his family and friends, going to different events and parties as he is invited. As he spends time with others, he is constantly having negative thoughts about himself and thinking about how everyone would react if they knew the horrible things he did while in the war. The memory of war makes it hard for him to cope. He believes that he is a bad person and assumes other people think he is, too. 

This is a borderline case because it exhibits the attributes of PTSD except for feelings of isolation and estrangement.

### 5.3. Contrary Case

Contrary cases exhibit what a concept is not [25]. They are beneficial to the analysis because it is simpler to determine what something is not rather than what it is. Acknowledging what a concept is not allows us to differentiate between the concept and the contrary case, providing insight into what defining attributes the concept should possess. 

A.B. is a veteran who frequently seeks mental health help at the local clinic in his hometown. He sees a psychiatrist once a month to talk about any issues he may have. A.B. is very forthcoming about his traumatic experiences while in the military and loves to participate in group therapy sessions to gain knowledge from other veterans. Whenever there are any events related to PTSD held at the clinic, A.B. always attends. He feels the more exposure he gains, the better he can manage his mental health issues. 

This is a contrary case because it does not exhibit any of the attributes of PTSD, intrusive memories of a traumatic event, feelings of isolation and estrangement, and negative cognitions.

## 6. Antecedents and Consequences

### 6.1. Antecedents

Events that must occur before the occurrence of a concept are antecedents [25]. A life-threatening or traumatic event, whether it is physical, mental, or emotional, must occur before the effects of PTSD can manifest. Merely witnessing or learning about an event that involves actual or threatening death, serious injury, or sexual violation can lead to the development of PTSD [8,37]. PTSD is induced by an external causative agent and is described as a mental health disorder following a traumatic event [38]. It is recognized as a clinical phenomenon that only happens after exposure to a traumatic event [24]. After this exposure, most people can recover from the initial symptoms. People who are not able to and continue to exhibit symptoms for at least one month may be diagnosed with PTSD [39]. There is a predisposition to PTSD based on how people process the event.

Trauma can be a difficult or unpleasant experience that causes someone to have mental or emotional problems, usually for a long time [27]. Clearly, trauma has the potential to harm an individual’s mental well-being and can lead to undesired health consequences. Seventy percent of people worldwide reported some form of exposure to a traumatic event [40]. Combat exposure and military sexual trauma are two traumatic events that the veteran may be exposed to [41]. Military veterans who serve in combat roles are at a greater risk of developing mental health problems [42]. There has been a link between combat exposure and PTSD among veteran men [5]. In addition, military sexual trauma, which refers to sexual assaults or harassment that occur during active duty, is more prevalent among women veterans. In women, the prevalence rate was 38.4% compared to 3.9% in men [43]. Also, women veterans who experienced military sexual trauma are more likely to have PTSD. Individuals who have undergone prior traumatic experiences without developing PTSD are vulnerable to developing the disorder in the future [17]. There is a significant correlation between a higher PTSD Checklist for DSM-5 scores among service personnel who sought help for mental health concerns five years after leaving the military compared to those who sought help within a period of less than five years [44,45].

### 6.2. Consequences

Consequences refer to the events that occur as the outcomes of the concept [25]. As a result of PTSD, patients have significant distress, cognitive impairment, and social/occupational dysfunction [1]. PTSD is a chronic condition that can be life-altering and disabling. It affects the overall well-being of patients and is the third most common disability in military veterans [3]. PTSD results in poor individual-level outcomes that include depression, substance abuse, and physical health problems [24]. Notably, PTSD and substance abuse frequently occur together [46]. Intrusive memories of traumatic events may lead to sleep and concentration difficulties [4]. This can remain with the person for decades or even a lifetime, provoking panic, terror, and despair [46]. In addition, PTSD causes emotional numbing, which leads to the avoidance of responsibilities and relationships [35]. Also, hyperarousal symptoms arise from restlessness and paranoia of PTSD, which result in other physiological stresses [6]. The stresses of PTSD can lead to tachycardia, increased blood pressure, tachypnea, and excessive sweating [47].

## 7. Empirical Referents

Empirical referents are how defining attributes are measured [25]. To measure the defining attributes of PTSD in veteran populations and assess the severity, structured interviews and self-report questionnaires are commonly used [2].

### 7.1. Primary Care PTSD Screen for DSM-5 (PC-PTSD-5)

The Primary Care PTSD Screen for DSM-5 (PC-PTSD-5) is a five-question screening method that most adequately measures the attributes of PTSD in veterans [48]. It begins with an initial assessment of any lifetime exposure to a traumatic event. If the answer is no, the screening is complete. If the participant answers yes, five additional questions inquire about how that trauma exposure has affected them in the past month. Any positive results from this screening do require further assessment from a structured interview or validated self-reported measures [48].

### 7.2. Clinician-Administered PTSD Scale for DSM-5 (CAPS-5)

The Clinician-Administered PTSD Scale for DSM-5 (CAPS-5) is considered the gold standard for PTSD assessment in military veterans [11,49,50]. It assesses for the DSM-5 PTSD diagnostic status and symptom severity [49]. There are three versions of the CAPS-5 that can correspond to different time periods: past week, past month, and worst month. The severity rating is from 0 (absent) to 4 (extreme/incapacitating) [51]. It is designed to be only administered by clinicians and clinical researchers with a working knowledge of PTSD or by a trained paraprofessional. This method of assessing PTSD is time-consuming, taking up to an hour to administer [51].

### 7.3. PTSD Checklist for DSM-5 (PCL-5)

The PTSD Checklist for DSM-5 (PCL-5) is a self-report measure, using a five-point Likert scale, that assesses the severity of the 20 PTSD symptoms during the last month [13]. It was created to be in alignment with how the criteria in diagnosing PTSD had changed and attempted to clarify symptoms [52]. With this tool, PTSD symptoms can be measured directly. Veterans being treated for PTSD by the Department of Veterans Affairs are required to complete it as part of a national initiative to measure the outcomes of their treatment [2]. Being one of most widely used tools to measure PTSD, scores of 31–33 are ideal for determining probable PTSD [49]. PCL-5 is one of the few measurement tools that are both up to the standard with the DSM-5 and have psychometric properties [50]. There are some barriers with this self-report system as participants may tend to over-report/under-report to achieve compensation or to avoid the stigma of PTSD [53].

## 8. Discussion

PTSD is one of the most common disabilities in veterans [3]. However, most veterans with PTSD do not receive mental healthcare [15,53]. Clearly, understanding the concept of PTSD in veterans is critically important. Hence, this study sought to analyze the concept of PTSD among the veteran population. The strength of this concept analysis is that it targets the main issues that veterans with PTSD deal with. Providing clear defining attributes of the concept of PTSD can help healthcare providers develop effective assessment strategies and interventions for veterans. 

Sekhon performed a concept analysis on PTSD using Walker and Avant’s method from a nursing perspective, suggesting the five attributes of a triggering event or events, re-experiencing, fear, helplessness, and loss or lack of meaning [31]. Similarly, Nayback conducted a concept analysis of PTSD using Walker and Avant’s method indicating the attributes of re-experiencing, hyperarousal, and avoidance [35]. However, there is still a lack of concept analysis on PTSD in veterans. Accordingly, this concept analysis of PTSD focused on the veteran population. The results showed that the defining attributes of PTSD are intrusive memories of traumatic events, feelings of isolation and estrangement, and negative cognitions. Re-experiencing is a common theme in both the previous and current analysis, suggesting that this symptom is central to PTSD. The findings from this concept analysis show the complexity and multifaceted nature of the disorder in veterans. Hence, understanding each aspect of these attributes of PTSD among veterans can help clinicians assess patient histories of traumatic events and guide treatment. Increased awareness of the struggles and difficulties that are related to PTSD and military service can be combated with prompt referrals to a mental health professional [54].

Many veterans with PTSD do not access mental health services [15,53]. Recent studies by Hundt et al. [18] and Johnson and Possemato [19] indicated that lack of knowledge is the most reported barrier to veterans seeking treatment. Not knowing or understanding the concepts of PTSD can further impede the veteran population from seeking treatment as well as affect how clinicians care for them. Also, it is necessary for nonveteran mental health and medical providers to be educated and trained to ensure they are able to offer appropriate and effective strategies for interacting with veterans [55].

In addition, gender may influence treatment-seeking behavior among veterans. A longitudinal study conducted by Harper et al. aimed to provide clarity on the associations between PTSD, intimate relationship functioning, and mental health service use for male and female veterans [14]. The findings revealed that poorer intimate relationship functioning related to PTSD severity had a positive effect on male veterans, but not women, in seeking treatment for PTSD. However, women veterans in general were more likely to seek treatment than men [14]. Moreover, appropriate peer support significantly impacts PTSD symptoms, aiding veterans in better managing them [54,56,57]. This suggests that isolation and estrangement play a critical role in the treatment-seeking behaviors of veterans. Understanding this dynamic is essential in tailoring interventions and support systems that address these needs. 

Furthermore, Ebadi et al. conducted a concept analysis on the treatment adherence of war veterans with PTSD using Walker and Avant’s method [58]. As PTSD relates to treatment adherence, the main attributes that arose were characteristics of PTSD, the risk factors for trauma, the characteristics of treatment plans, and the characteristics of healthcare teams. PTSD type, severity, and manifestations can predict treatment nonadherence [58]. An individual with chronic PTSD has persistent symptoms over an extended period of time and may struggle with treatment adherence. PTSD symptom severity makes it challenging for an individual to adhere to treatment due to the overwhelming difficulties in managing life [57]. Providing therapeutic follow-up services, being available for patients, communication with patients, and client–provider conflicts can influence treatment adherence [58]. When a treatment plan is complex and hard to manage, it will likely lead to nonadherence. Hence, PTSD-related factors and treatment-related factors should be considered for promoting treatment adherence. 

The symptoms of PTSD can significantly impact an individual’s quality of life and contribute to other mental health issues and a wide variety of medical issues, from pain to cardiovascular problems [59]. The findings of this concept analysis revealed that the consequences of PTSD include physical health problems, cognitive impairment, social/occupational dysfunction, and other mental health disorders. Thus, the appropriate and effective treatment of the main attributes of PTSD is essential. Notably, the symptoms of PTSD in veterans can be alleviated by prompt diagnosis and treatment if they are diagnosed and treated in a timely manner [59]. A randomized control trial, conducted by Schnurr and Lunney in 235 female veterans and soldiers, examined residual PTSD symptoms after 10 weekly sessions of Prolonged Exposure (PE) and Present-Centered Therapy (PCT) [60]. The Clinician-Administered PTSD Scale (CAPS) was used to assess PTSD symptoms at baseline and one week after the completion of sessions. Although both treatments showed a reduction in symptoms, conditional probabilities of retaining intrusive memories, avoidance of people and places, feelings of detachment and estrangement, and restricted range of affect were lower in PE than PCT [60]. Despite evidence-based treatment options, women veterans continue to report a higher prevalence of PTSD compared to both civilian women and men veterans [15].

Another study with a randomized waitlist-controlled design sought to test the efficacy of the reconsolidation of traumatic memories (RTM) among a group of male veterans who had recent flashbacks and nightmares within the current month [61]. The participants were randomly assigned to the RTM treatment group or the untreated waitlisted control group. For the between-group comparison, the findings showed that there were significant differences between the treatment group at two weeks post-treatment and the untreated waitlisted control group at the end of the wait period. It suggested that the RTM treatment may be an effective intervention in improving PTSD symptoms for veterans [61].

Horwitz et al. assessed the relationship between negative post-traumatic cognitions and suicidal ideation in 177 veterans and service members who completed a three-week intensive outpatient treatment program for PTSD [34]. The results indicated that negative post-traumatic cognitions, specifically negative thoughts about oneself, had a significant association with suicidal ideation after controlling for the severity of PTSD, depression, and pretreatment suicidal ideation. In addition, PTSD can negatively impact the status of intimate relationships leading to isolation and feelings of estrangement [14]. Notably, with the reduction in self-blame and negative cognitions, PTSD symptomology can be managed and changed [34]. The current analysis highlights the importance of recognizing the impact that negative cognitions have on the recovery of PTSD. Hence, it is necessary to improve maladaptive emotional regulation strategies to reduce the prevalence of PTSD and symptoms of suicidal ideation, and increase recovery [62].

## 9. Conclusions

The concept of PTSD was analyzed using the modified method outlined by Walker and Avant [25]. Three defining attributes are identified for the concept of PTSD in veterans: intrusive memories of traumatic events, feelings of isolation and estrangement, and negative cognitions. PTSD dramatically changes the lives of those who suffer from it. A better understanding of the concept of PTSD can facilitate the development of effective interventions for the veteran population and enhance their mental health. These attributes highlight the interplay between cognitive, emotional, and social factors. Understanding these key components allows us to customize interventions that meet the unique needs of veterans with PTSD and ultimately improve their overall health and well-being. Veterans themselves could benefit from comprehending the concepts of PTSD, as it allows them to recognize their symptoms, seek appropriate support, and actively engage in their recovery. Continued research aimed at refining the attributes of PTSD is essential for healthcare providers in fostering resilience in this vulnerable population. 

## Data Availability

Not applicable.
